# Supplementary material for: Captivity and the co-diversification of great ape microbiomes
Source: Nat Commun. 2021 Sep 24;12:5632. doi: 10.1038/s41467-021-25732-y (PMC8463570; doi:10.1038/s41467-021-25732-y)
Supplement: Supplementary file 1 — Supplementary information [file 41467_2021_25732_MOESM1_ESM.pdf]

Supplemental information

Captivity and the Co-diversification of Great Ape Microbiomes

Alex H. Nishida[1,2*], Howard Ochman[1,2]

[1] Department of Integrative Biology,

The University of Texas at Austin, Austin, TX, USA. [2] Molecular Biosciences, The University of Texas at Austin, Austin, TX, USA. email: ahnishida@utexas.edu

Additional program and packages

Select fastqs were cleaned with repair.sh from bbmap[1]. Fastqs were demultiplexed with either demultiplex[2] or barcode-splitter[3] depending on barcode format. Sequences were translated and aligned with transeq and transalign from EMBOSS[4] and identity was assessed using BLAST[5]. General data processing, statistical analyses, and visualization in R were accomplished with the following packages: tidyverse[6], cowplot[7], ggtree[8], RColorBrewer[9], statix[10], and reshape2[11], genefilter[12], seqinr[13], ape[14], phytools[15], RVAideMemoire[16], broom[17], and zoo[18]. Data processing in python was accomplished with pandas[19], numpy[20], and Biopython[21].

Supplementary References

1. Bushnell, B. BBMap: a fast, accurate, splice-aware aligner. https://jgi.doe.gov/data-and-tools/bbtools (2014).
2. Laros, J. F. J. demultiplex: demultiplex FASTA or a FASTQ files based on a list of barcodes. https://pypi.org/project/demultiplex/0.1.1/ (2018).
3. Leach, R. & Parsons, L. barcode-splitter: a utility to split multiple sequence files using multiple sets of barcodes. https://pypi.org/project/barcode-splitter/ (2017).
4. Bininda-Emonds, O. R. P. transAlign: using amino acids to facilitate the multiple alignment of protein-coding DNA sequences. *BMC Bioinformatics* **6**, 1–6 (2005).
5. Altschup, S. F., Gish, W., Miller, W., Myers, E. W. & Lipman, D. J. Basic Local Alignment Search Tool. **215**, 403–410 (1990).
6. Wickham, H. *et al.* Welcome to the Tidyverse. *J. Open Source Softw.* **4**, 1686 (2019).
7. Wilke, C. O. cowplot: streamlined plot theme and plot annotations for 'ggplot2'. https://cran.r-project.org/web/packages/cowplot/cowplot (2019).
8. Yu, G., Lam, T. T.-Y., Zhu, H. & Guan, Y. Two methods for mapping and visualizing associated data on phylogeny using ggtree. *Mol. Biol. Evol.* **35**, 3041–3043 (2018).
9. Neuwirth, E. RColorBrewer: ColorBrewer Palettes. https://cran.r-project.org/web/packages/RColorBrewer/index.html (2014).
10. Kassambara, A. rstatix: pipe-friendly framework for basic statistical tests. https://cran.r-project.org/web/packages/rstatix/index.html. (2021).
11. Wickham, H. Reshaping Data with the reshape Package. *J. Stat. Softw.* **21**, 1–20 (2007).
12. Gentleman, R., Carey, V. J., Huber, W. & Hahne, F. genefilter: methods for filtering genes from high-throughput experiments. https://bioconductor.org/packages/release/bioc/html/genefilter.html (2021).
13. Charif, D. & Lobry, J. R. SeqinR 1.0-2: a contributed package to the R project for statistical computing devoted to biological sequences retrieval and analysis. in *Structural approaches to sequence evolution: Molecules, networks, populations* (eds. Bastolla, U., Porto, M., Roman, H. E. & Vendruscolo, M.) 207–232 (Springer Verlag, 2007).
14. Paradis, E., Claude, J. & Strimmer, K. APE: analyses of phylogenetics and evolution in R language. *Bioinformatics* **20**, 289–290 (2004).
15. Revell, L. J. phytools: an R package for phylogenetic comparative biology (and other things). *Methods Ecol. Evol.* **3**, 217–223 (2012).
16. Hervé, M. RVAideMemoire: testing and plotting procedures for biostatistics. https://cran.r-project.org/package=RVAideMemoire (2020).
17. Robinson, D. broom: convert statistical objects into tidy tibbles. https://cran.r-project.org/web/packages/broom/index.html (2014).
18. Zeileis, A., Grothendieck, G., Ryan, J. & Andrews, F. zoo: S3 infrastructure for regular and irregular time series. https://cran.r-project.org/web/packages/zoo/index.html (2005).
19. Team, P. development. Pandas. doi:10.5281/zenodo.3509134 (2020).
20. Harris, C. R. *et al.* Array programming with NumPy. *Nature* **585**, 357–362 (2020).
21. Cock, P. J. A. *et al.* Biopython: freely available Python tools for computational molecular biology and bioinformatics. *Bioinformatics* **25**, 1422–1423 (2009).

Figure S1

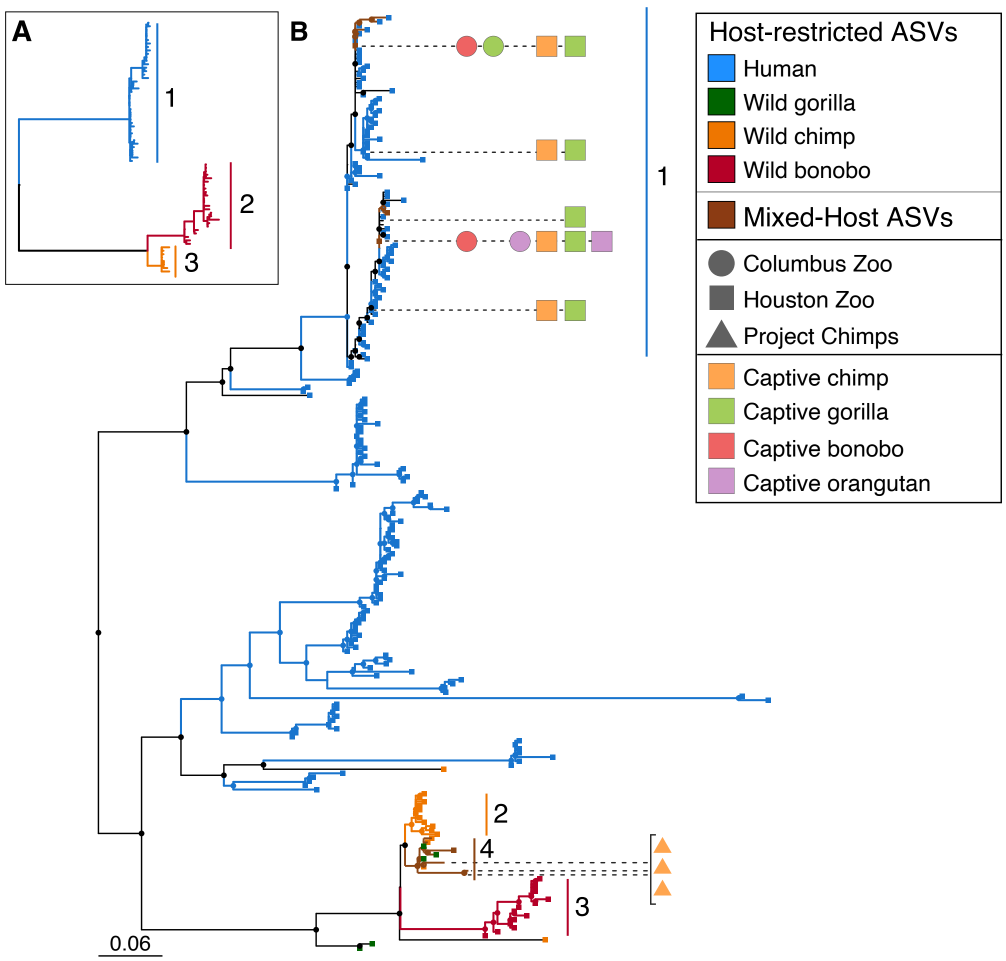

Figure S1. Phylogeny of newly identified ASVs belonging to co-diversified lineage Bacteroidaceae 3 (Bt3 in Figure 4). Inset (A) at upper left shows topology of the original co-diversified clades identified in Moeller *et al.*[9] with branches color-coded to denote host species. The three major clades from this inset tree (Clades 1-3) are highlighted and labelled in the phylogeny that includes newly identified *gyrB*-ASVs (B). An additional mixed-host clade is also labeled (Clade 4). Lineages and terminal tips are color-coded to indicate the host-species source of an ASV, and dashed lines correspond to ASVs identified in captive apes. Nodes with bootstrap support >50 are indicated.

Figure S2

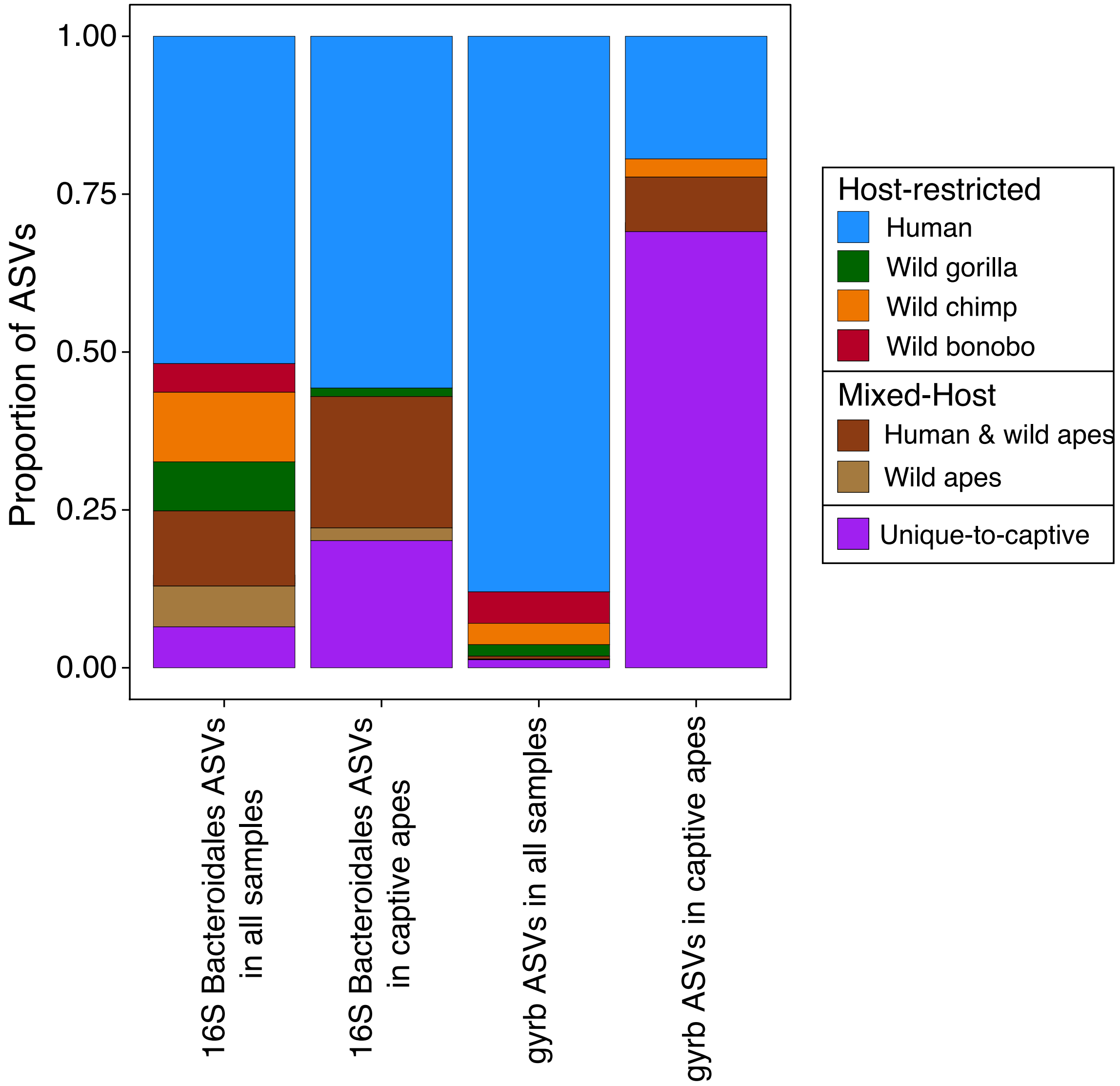

Figure S2. Proportions of host-restricted and mixed-hosted ASVs in 16S and *gyrB* datasets across all samples and within captive apes.

Figure S3

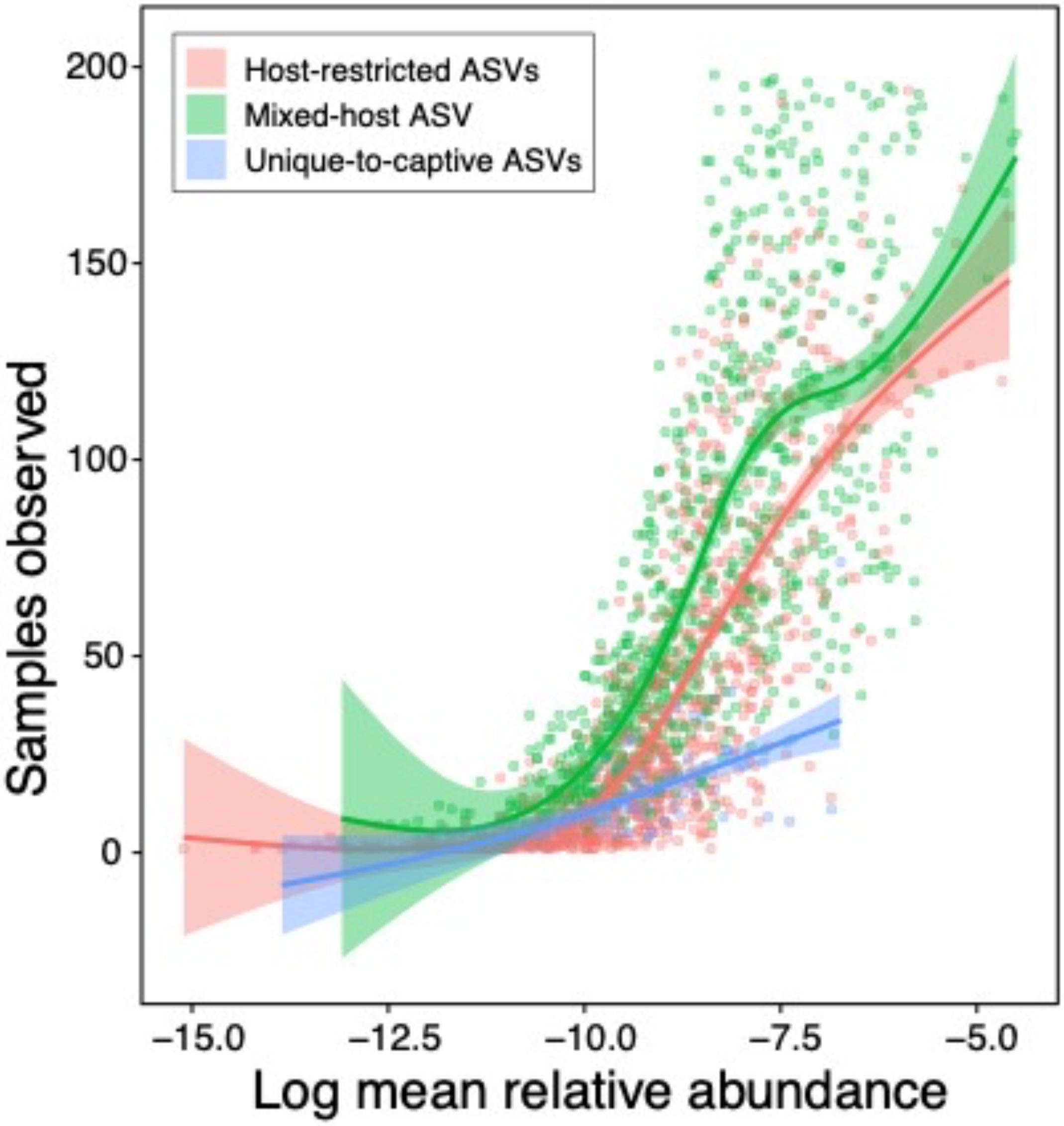

Figure S3. Relationship between ASVs abundance and sample occupancy. Generalized Additive Model showing the relationship between abundance and sample occupancy of 16S-ASVs observed in up to 200 host samples classified according to status as host-restricted (df = 5.95, $r^2 = 0.65$), mixed-host (df = 6.74, $r^2 = 0.59$), or unique-to-captive apes (df = 7.87, $r^2 = 0.36$) with shaded areas representing 95% confidence intervals.

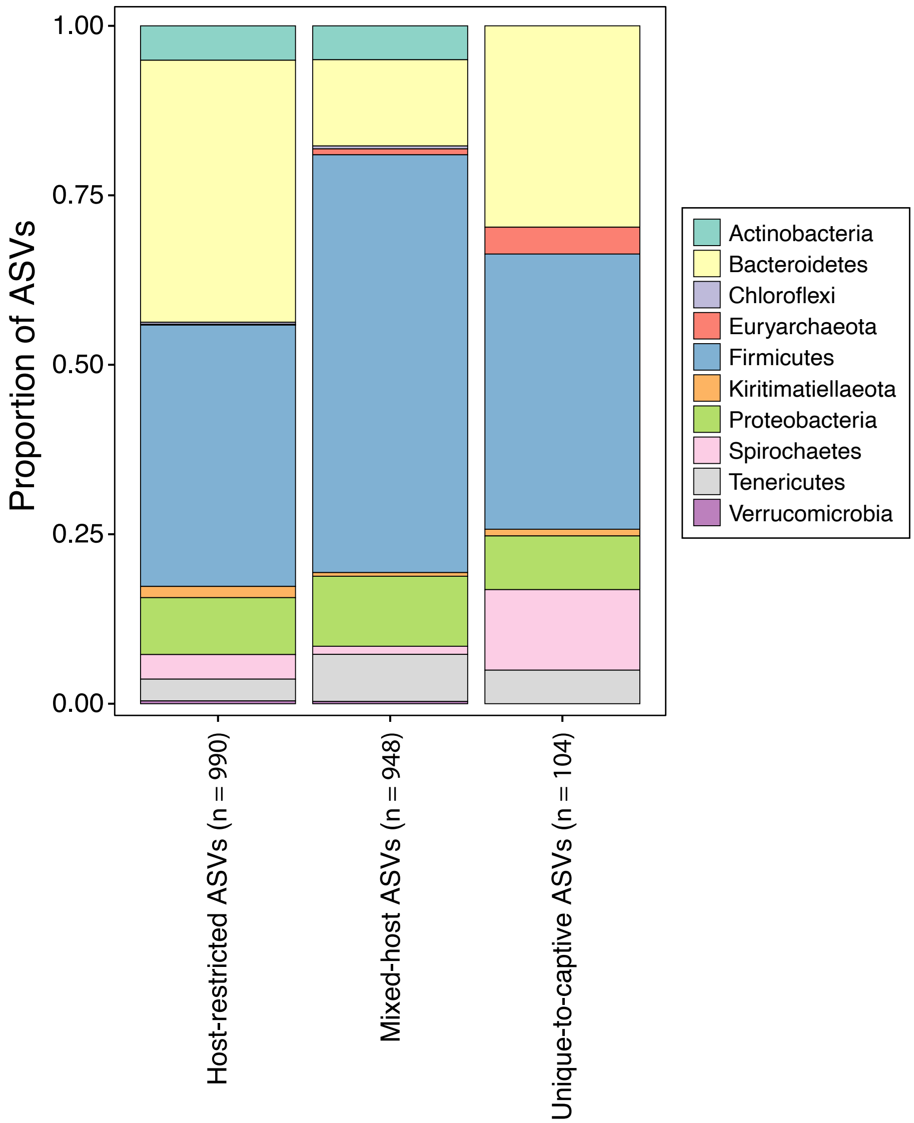

Figure S4. Proportions of host-restricted, mixed-host, and unique-to-captive-apes 16S-ASVs assigned to bacterial phyla. *n* = the total numbers of 16S-ASVs assigned to each category. Differences in the number of Bacteroidetes and Firmicutes 16S-ASVs designated as host-restricted and mixed-host contribute to 67% to the total Chi-square score [$X^2 = 255.93$ (df = 16, n = 2,042), $p < .0001$].

Figure S5

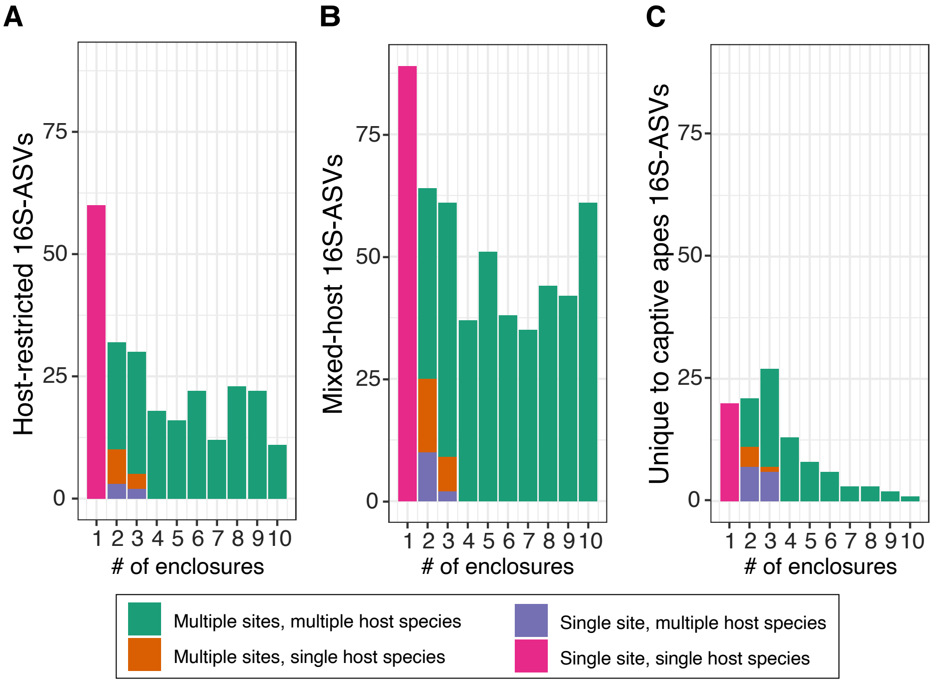

Figure S5. Distribution of (A) host-restricted, (B) mixed-host, and (C) unique-to-captive-apes 16S-ASVs across zoo enclosures. ASVs are color-coded according to their presence in single or multiple host-species and/or zoo sites. (Note that all zoos maintain host species in separate enclosures, therefore ASVs observed in a single enclosure are, by default, observed in a single host species and zoo site.)

Figure S6

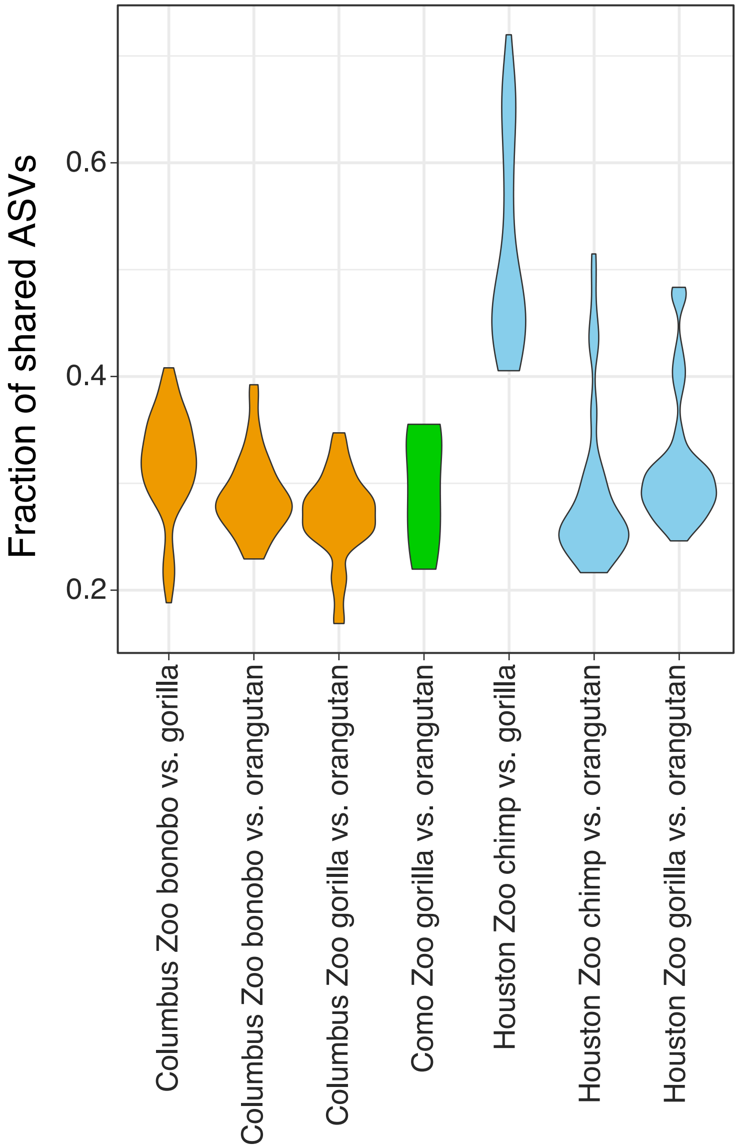

Figure S6. Comparisons of the proportions of shared 16S-ASVs (Sørenson similarity index) among individuals residing in the same zoo but belonging to different host species.

Figure S7

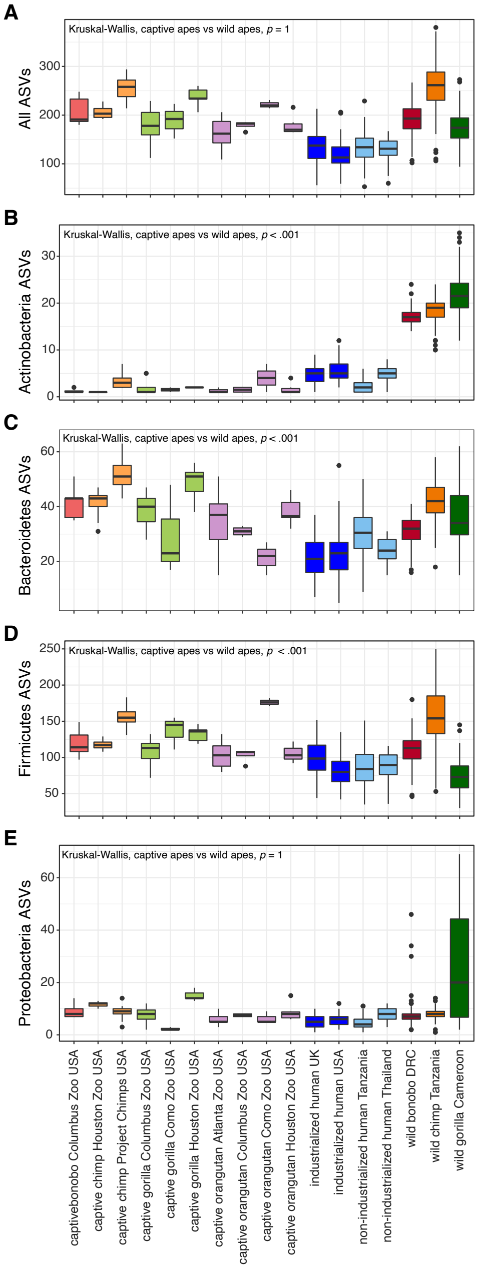

Figure S7. Alpha diversity of wild ape, captive ape, non-industrialized human, and industrialized human samples, measured by observed 16S-ASVs. (A) in all phyla, and (B–E) in the major phyla present in the gut microbiome. FDR-adjusted p-values of Kruskal-Wallis ranked sum tests between wild apes (n = 377) and captive apes (n = 77) are shown for all phyla (df = 1, H = .4, p = 1), Actinobacteria (df = 1, H = 192.4, p = 4.710e-43), Bacteroidetes (df = 1, H = 14.5, p = 6.900e-04), Firmicutes (df = 1, H = 17.9, p = 1.185e-04) , and Proteobacteria (df = 1, H = 1.5, p = 1). Boxes display the median and IQR (25th & 75th percentiles) and whiskers represent the minimum and maximum values within 1.5xIQR of the upper and lower quartiles for each sample group.

Figure S8

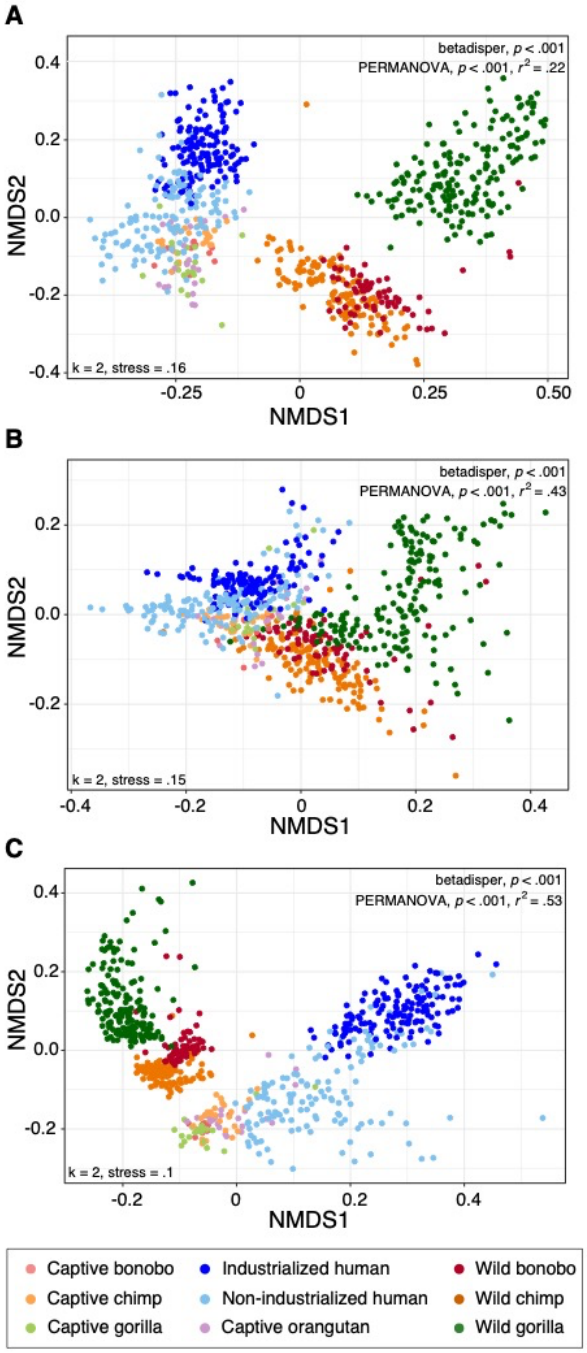

Figure S8. Gut microbiome composition of captive great apes, wild great apes, and humans visualized by non-metric multidimensional scaling (NMDS). Ordination based on (A) Jaccard distances (PERMANOVA, df = 8, F = 25.9, p = .001, r^2 = .22; betadisper, df = 8, F = 50.0, p = .001), (B) weighted UniFrac distances (PERMANOVA, df = 8, F = 68.2, p = .001, r^2 = .43; betadisper, df = 8, F = 23.2, p = .001), and (C) unweighted UniFrac distances (PERMANOVA, df = 8, F = 102.6, p = .001, r^2 = .53; betadisper, df = 8, F = 81.8, p = .001)

Figure S9

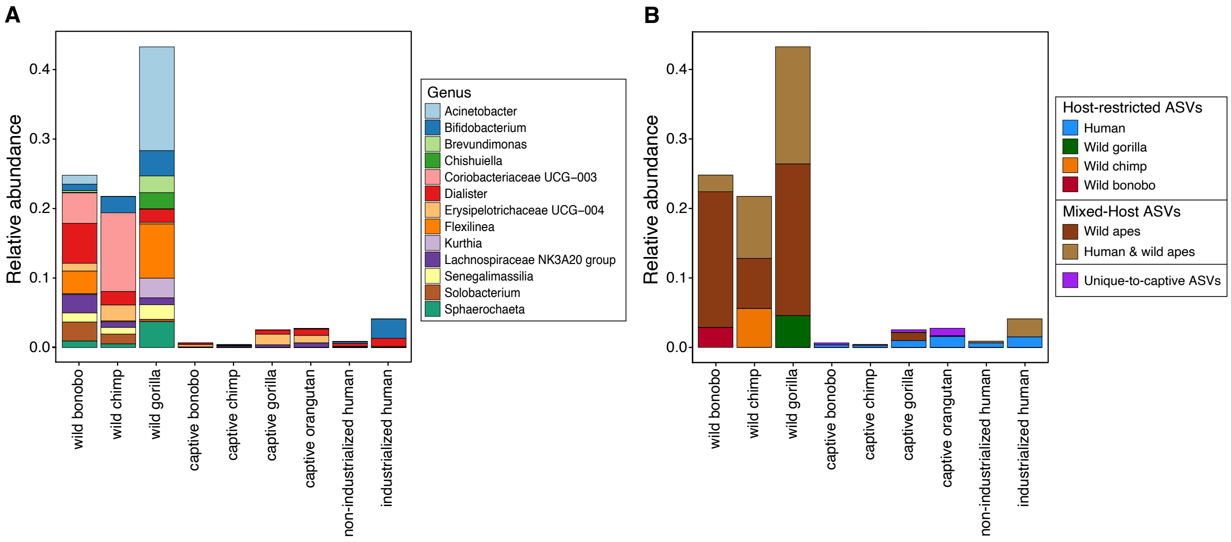

Figure S9. Average relative abundances of ASVs showing depletion in captive apes. (A) Average relative abundances of microbial genera that are concurrently depleted in captive great ape species relative to wild great ape species. (B) Average relative abundances of host-restricted 16S-ASVs, mixed-hosted 16S-ASVs, and unique-to-captive apes 16S-ASVs, within genera depleted in captive apes.

Figure S10

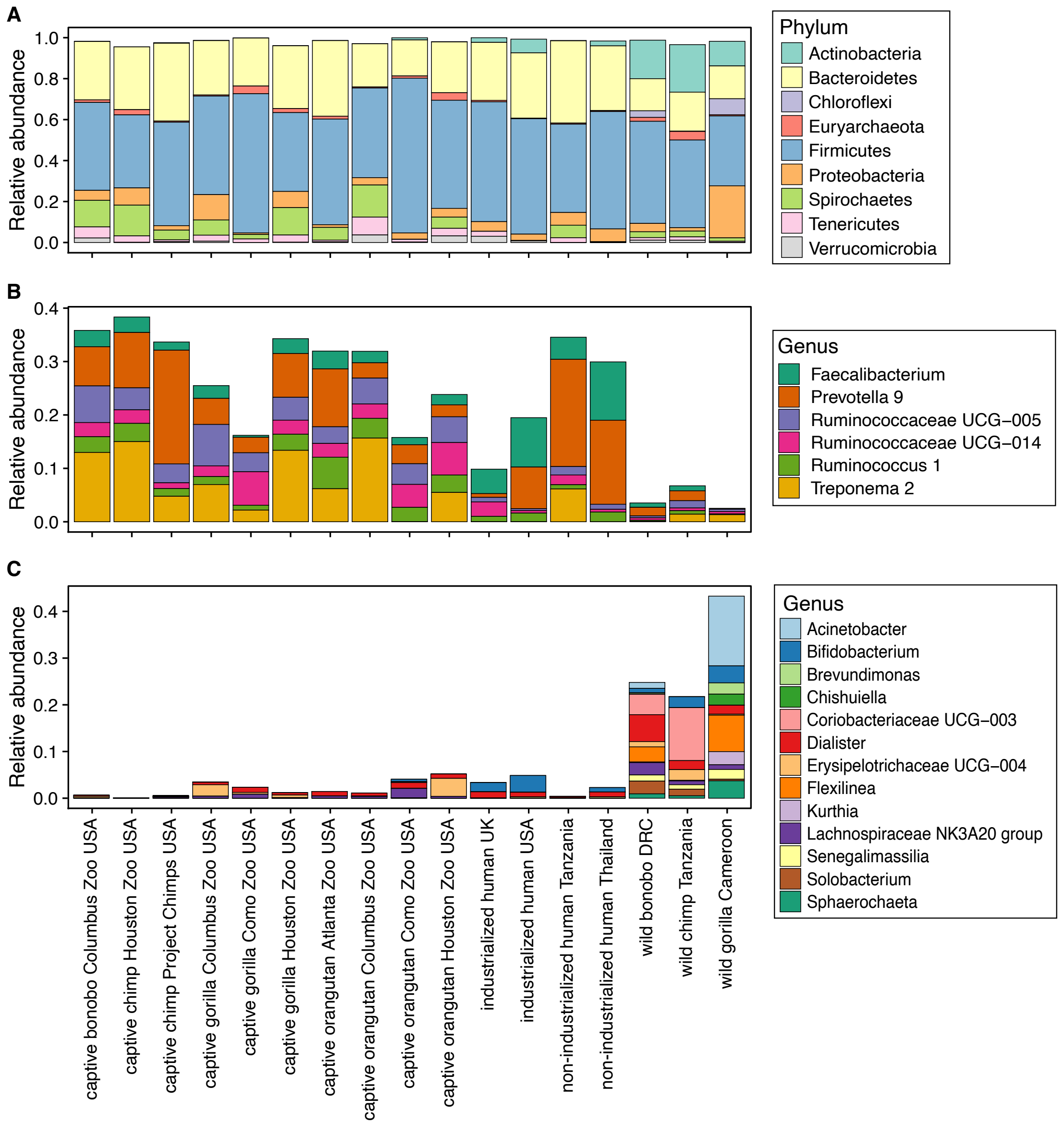

Figure S10. Average relative abundances of ASVs across hosts species according to geographic site. (A) Average relative abundances of bacterial phyla across captive apes, wild apes and humans sorted according to geographic site. (B) Average relative abundances of bacterial genera enriched in captive great ape species relative to wild great ape species. (C) Average relative abundances of bacterial species depleted in captive great ape species relative to wild great ape species.
