# Supplementary material for: Captivity and the co-diversification of great ape microbiomes
Source: Nat Commun. 2021 Sep 24;12:5632. doi: 10.1038/s41467-021-25732-y (PMC8463570; doi:10.1038/s41467-021-25732-y)
Supplement: Supplementary file 3 — Description of Additional Supplementary Files [file 41467_2021_25732_MOESM3_ESM.docx]

**Description of Additional Supplementary Files**

File Name: Supplemental data 1

Description: Metadata for 16S amplicon samples

File Name: Supplemental data 2

Description: Metadata for gyrb amplicon and metagenomic samples

File Name: Supplemental data 3

Description: Individual histories and dietary information for captive great apes provided by the Houston and Columbus Zoos

File Name: Supplemental data 4

Description: 16S and gyrB forward and reverse primers

File Name: Supplemental data 5

Description: Results of betadisper and PERMANOVA comparing microbiome compositions of great apes and humans based on captivity status and host species

File Name: Supplemental data 6

Description: Mean relative abundances of bacterial genera and results of Kruskal-Wallis rank sum tests among captive apes and wild apes
