# Supplementary material for: Captivity and the co-diversification of great ape microbiomes
Source: Nat Commun. 2021 Sep 24;12:5632. doi: 10.1038/s41467-021-25732-y (PMC8463570; doi:10.1038/s41467-021-25732-y)
Supplement: Supplementary file 10 — Reporting Summary [file 41467_2021_25732_MOESM10_ESM.pdf]

nature research

Corresponding author(s): Alex H Nishida

Last updated by author(s): 2021/08/13

Reporting Summary

Nature Research wishes to improve the reproducibility of the work that we publish. This form provides structure for consistency and transparency in reporting. For further information on Nature Research policies, see our Editorial Policies and the Editorial Policy Checklist.

Statistics

For all statistical analyses, confirm that the following items are present in the figure legend, table legend, main text, or Methods section.

n/a	Confirmed	
☐	☒	The exact sample size (*n*) for each experimental group/condition, given as a discrete number and unit of measurement
☐	☒	A statement on whether measurements were taken from distinct samples or whether the same sample was measured repeatedly
☐	☒	The statistical test(s) used AND whether they are one- or two-sided<br>*Only common tests should be described solely by name; describe more complex techniques in the Methods section.*
☐	☒	A description of all covariates tested
☐	☒	A description of any assumptions or corrections, such as tests of normality and adjustment for multiple comparisons
☐	☒	A full description of the statistical parameters including central tendency (e.g. means) or other basic estimates (e.g. regression coefficient) AND variation (e.g. standard deviation) or associated estimates of uncertainty (e.g. confidence intervals)
☐	☒	For null hypothesis testing, the test statistic (e.g. *F*, *t*, *r*) with confidence intervals, effect sizes, degrees of freedom and *P* value noted<br>*Give P values as exact values whenever suitable.*
☒	☐	For Bayesian analysis, information on the choice of priors and Markov chain Monte Carlo settings
☒	☐	For hierarchical and complex designs, identification of the appropriate level for tests and full reporting of outcomes
☒	☐	Estimates of effect sizes (e.g. Cohen's *d*, Pearson's *r*), indicating how they were calculated

Our web collection on statistics for biologists contains articles on many of the points above.

Software and code

Policy information about availability of computer code

Data collection	Code and processed data to generate all figures and tables are available in a dedicated Github repository (https://github.com/ahnishida/captive_ape_microbiome, doi: 10.5281/zenodo.5188501)
Data analysis	Re-pair reads in disordered fastq (repair.sh from bbmap v38.70)<br>Demultiplex fastqs with barcode-splitter(v0.18.6) or demultiplex(v1.0.1)<br>Remove primers with cutadapt(v2.5)<br>Read trimming, error correction, chimera removal and generate ASV table with DADA2(v1.16.0)<br>Align sequences with mafft(v7.309)<br>Generate ASV phylogenies with FastTree(v2.1.9)<br>General data processing in R with tidyverse(v1.3.0), reshape2(v1.4.4), rstatix(v0.6.0)<br>Processing microbiome datasets in R with phyloseq(v1.32.0), DECIPHER(v2.16.1), genefilter(v1.70.0), seqinr(v3.6-1), ape(v5.4-1), phytools (v0.7-70), zoo(v1.8-8), picante(v1.8.2)<br>Sequence analysis with prodigal(v2.6.3), hmmer(v3.3), transeq and transalign from EMBOSS(v6.6.0.0), BLAST(v2.9.0)<br>General data processing in python with pandas(v1.0.5), numpy(v1.19.0)<br>Sequence analysis and defining HR clades in python with Biopython(v1.77), ete3(v3.1.1)<br>Statistical analysis in R with PMCMRplus(1.5.1), RVAideMemoire(v0.9-78), broom(0.7.1), Vegan(v2-5.6)<br>Data visualization in R with ggplot2(v3.3.3), ggtree(v2.2.4), cowplot(v1.1.0), RColorBrewer(v1.1-2)

For manuscripts utilizing custom algorithms or software that are central to the research but not yet described in published literature, software must be made available to editors and reviewers. We strongly encourage code deposition in a community repository (e.g. GitHub). See the Nature Research guidelines for submitting code & software for further information.

Data

Policy information about availability of data

All manuscripts must include a data availability statement. This statement should provide the following information, where applicable:

- Accession codes, unique identifiers, or web links for publicly available datasets
- A list of figures that have associated raw data
- A description of any restrictions on data availability

16S-amplicon and gyrB-amplicon sequence data produced by this study are publicly available under accession numbers (PRJNA692991; PRJNA693013), and we used the accession numbers from published studies to acquire additional data.

Field-specific reporting

Please select the one below that is the best fit for your research. If you are not sure, read the appropriate sections before making your selection.

[x] Life sciences [ ] Behavioural & social sciences [ ] Ecological, evolutionary & environmental sciences

For a reference copy of the document with all sections, see nature.com/documents/nr-reporting-summary-flat.pdf

Life sciences study design

All studies must disclose on these points even when the disclosure is negative.

Sample size	Sample sizes are based on the number of captive apes housed in enclosures at US zoos. We included human and wild ape samples from previously published studies, and these data were sampled at roughly equivalent numbers.
Data exclusions	Samples were excluded from the analysis if they failed to meet the 10,000 read rarefaction threshold.
Replication	In library preparation, samples were PCR reactions were performed in triplicate and successful reactions were merged.
Randomization	Not applicable for an observational microbiome study because there is no treatment assignment in this study.
Blinding	Not applicable for an observational microbiome study because there is no treatment assignment in this study.

Reporting for specific materials, systems and methods

We require information from authors about some types of materials, experimental systems and methods used in many studies. Here, indicate whether each material, system or method listed is relevant to your study. If you are not sure if a list item applies to your research, read the appropriate section before selecting a response.

Materials & experimental systems

n/a	Involved in the study	
x		Antibodies
x		Eukaryotic cell lines
x		Palaeontology and archaeology
	x	Animals and other organisms
x		Human research participants
x		Clinical data
x		Dual use research of concern

Methods

n/a	Involved in the study	
x		ChIP-seq
x		Flow cytometry
x		MRI-based neuroimaging

Animals and other organisms

Policy information about studies involving animals; ARRIVE guidelines recommended for reporting animal research

Laboratory animals	This study did not involve laboratory animals
Wild animals	Fecal samples were collected through noninvasive means from captive great apes, the species, sex, and age of the individuals are available in Table S3.
Field-collected samples	Samples collected with 12 hours of deposition and were stored in RNAlater at -20 degrees and shipped overnight to laboratory for DNA extraction.
Ethics oversight	Research proposal were submitted to individual zoos for review by zoo staff to determine compliance with the Association of Zoos and Aquariums.

Note that full information on the approval of the study protocol must also be provided in the manuscript.
